# Quo vadis precision oncology?

**DOI:** 10.1007/s00432-022-04478-0

**Published:** 2023-01-13

**Authors:** Kathrin Heinrich, C. Benedikt Westphalen

**Affiliations:** 1 Comprehensive Cancer Center Munich & Department of Medicine III, University Hospital Munich, LMU Munich, Marchioninistr. 15, 81377 Munich, Germany; 2 German Cancer Consortium (DKTK Partner Site Munich), Heidelberg, Germany

Precision oncology aims to deliver individualized therapeutic interventions to cancer patients. As of today, precision oncology is defined by the integration of genomic tumor profiling into clinical decision-making. With the rapidly growing number of (histology-agnostic) molecularly guided therapy options (MGTO), precision oncology is gaining tremendous momentum (Mateo et al. 2022). Looking into an exciting future, it is time to reflect on aspects of the past and to assess the present situation.

More than 20 years ago, with the advent and breathtaking success of imatinib for Bcr-abl positive chronic myeloid leukemia (Druker et al. 2001a, 2001b), the prospect of specifically targeting the molecular roots of a given malignancy became reality. With a rapidly growing understanding of the molecular foundations of cancer on one side and the growing number of targeted agents on the other side, the hope to push targeted cancer therapy into treatment reality for many cancers was fueled.

While spectacular success has been made in the targeted treatment of some malignancies, most prominently non-small cell lung cancer (Thai et al. 2021), implementation of precision oncology in other solid malignancies has been somewhat slower.

There are countless reasons for this observation reaching from the complexity of cancer biology to the design of clinical trials and finally limited access to innovative agents. Discussing these complexities would be beyond the scope of this editorial.

Here, we would like to focus on one specific aspect, namely the use of genomic tumor profiling in later stage cancers. Multiple clinical trials have tried to test the concept of allocation of targeted agents based on molecular markers identified using genomic profiling. The most prominent early trials, SHIVA (Tourneau et al. 2014) and PROFILER (Tredan et al. 2019), failed to show a benefit of this approach and MOSCATO (Massard et al. 2017) delivered modest clinical benefit for a small subset of patients. Similarly, multiple cohorts within the NCI-MATCH trial failed their modest thresholds for being deemed successful. This led some investigators to the conclusion that the whole concept of genomics-driven precision oncology was futile (Tannock and Hickman 2019). However, it was not considered that these earlier clinical trials included mostly patients with very advanced disease and the targeted treatment options available at the time of recruitment would now be considered suboptimal.

At the same time, dedicated precision oncology programs and molecular tumor boards became clinical reality in cancer centers around the globe. With careful patient selection, structured access to molecular tumor boards, and clinical trial programs offering novel and innovative treatment options, new success stories began to emerge.

As of now, multiple targeted agents have been approved in a histology-agnostic fashion, meaning that the biomarker defines the indication for treatment independent of the underlying histology. At the same time, a broad variety of novel agents targeting ever more (genomic) biomarkers are in late clinical development offering the realistic hope that a large fraction of cancer patients will soon have targeted treatment options.

What does this mean for our health care system?

As of now, access to genomic tumor profiling is still limited, yet if we want our cancer patients to benefit from novel treatment options, access to innovation within clinical trials and to offer them the most comprehensive analyses of their individual cancers, we cannot limit access to diagnostics to few patients, in academic centers in privileged countries (Mateo et al. 2022).

This realization has broad socio-economic and ethical implications as barriers to diagnostics will have fundamental impact on the therapeutic management of cancer patients in the near future. More importantly, access to testing remains pointless, if access to innovative treatment inside and outside of clinical trials is lacking.

Accordingly, we, as the global cancer community, have the obligation to work on the concept of equity and access to carry the promise and hope of precision oncology forward.

## Data Availability

No primary data was used for this correspondence.
